# A Multistate Study on Housing Factors Influential to Heat-Related Illness in the United States

**DOI:** 10.3390/ijerph192315762

**Published:** 2022-11-26

**Authors:** Ming Hu, Kai Zhang, Quynh Camthi Nguyen, Tolga Tasdizen

**Affiliations:** 1 School of Architecture, Planning, Preservation, University of Maryland, College Park, MD 20742, USA; 2 Department of Environmental Health Sciences, School of Public Health, University at Albany, State University of New York, Rensselaer, NY 12144, USA; 3 Department of Epidemiology and Biostatistics, College Park School of Public Health, University of Maryland, College Park, MD 20742, USA; 4 Department of Electrical and Computer Engineering, Scientific Computing and Imaging Institute, University of Utah, Salt Lake City, UT 84112, USA

**Keywords:** housing factors, heat-related illness, thermal inertia, multistate

## Abstract

As climate change increases the frequency and intensity of devastating and unpredictable extreme heat events, developments to the built environment should consider instigating practices that minimize the likelihood of indoor overheating during hot weather. Heatwaves are the leading cause of death among weather-related causes worldwide, including in developed and developing countries. In this empirical study, a four-step approach was used to collect, extract and analyze data from twenty-seven states in the United States. Three housing characteristic categories (i.e., general housing conditions, living conditions, and housing thermal inertia) and eight variables were extracted from the American Housing Survey database, ResStock database and CDC’s National Environmental Public Health Tracking Network. Multivariable regression models were used to understand the influential variables, a multicollinearity test was used to determine the dependence of those variables, and then a logistic model was used to verify the results. Three variables—housing age (HA), housing crowding ratio (HCR), and roof condition (RC)—were found to be correlated with the risk of heat-related illness (HRI) indexes. Then, a logistic regression model was generated using the three variables to predict the risk of heat-related emergency department visits (EDV) and heat-related mortality (MORD) on a state level. The results indicate that the proposed logistic regression model correctly predicted 100% of the high-risk states for MORD for the eight states tested. Overall, this analysis provides additional evidence about the housing character variables that influence HRI. The outcomes also reinforce the concept of the built environment determined health and demonstrate that the built environment, especially housing, should be considered in techniques for mitigating climate change-exacerbated health conditions.

## 1. Introduction

According to the Intergovernmental Panel on Climate Change (IPCC) report, around 30% of the global population is exposed to extreme heat for at least 20 days each year [1]. Between 2000 and 2019, an average of six heat-related deaths per 100,000 residents each year was reported in North America [2]. According the World Health Organization, from 1998 to 2017, more than 166,000 people died due to heat waves, and between 2000 and 2016, the number of people exposed to heat waves increased by around 125 million [3]. More recently, 2021 experienced a record-breaking heat wave across North America [4], and according to the U.S. National Oceanic and Atmospheric Administration, August 2022 was the hottest August recorded in North America and Europe, and the second warmest August globally [5]. In the United States, the increase in extreme temperatures is expected to lead to a rise in heated-related deaths and illness, particularly for vulnerable populations and communities such as the elderly [6]. Most people stay indoors during heat events, thus, developments to the built environment should consider instigating housing design practices that minimize the likelihood of overheating during hot weather events. Indoor heat exposure can lead to a cascade of illnesses including heat exhaustion, heatstroke, and hyperthermia. In addition, extreme temperatures can worsen chronic conditions such as cardiovascular and respiratory diseases. Meanwhile, climate change is increasing the potential of devastating and unpredictable extreme heat events. The Climate Action Tracker states that the world is headed for 2.4 °C of warming, despite the COP26 climate pledges [7]. Under such conditions, it is imperative to prioritize the prevention of overheating in buildings. The first step is to understand what housing characteristics may have the biggest impact on heat-related illness (HRI).

According to the World Health Organization, heat waves rarely receive adequate attention because their death tolls and destruction are not always immediately obvious [3]. There are also different definitions for a heat wave. The U.S. Environmental Protection Agency defines a heat wave as a period of two or more consecutive days when the daily minimum apparent temperature (the actual temperature, adjusted for humidity) in a particular city exceeds the 85th percentile of historical July and August temperatures (1981–2010) for that city (refer to the EPA’s website for the reason for this definition) [8]. Extensive literature has focused on heat exposure in outdoor environments and its associated human health impacts, and the health impacts from extreme heat have used ambient meteorological measures [9]. However, the available data on indoor heat exposure and its effect on human health is relatively limited compared to that of outdoor heat exposure, and most studies are on a small scale (e.g., individual buildings, a group of homes) [10,11]. For example, Williams and colleagues conducted a study on low-income senior residents (n = 51) in public housing in Cambridge, Massachusetts. They found that with higher indoor temperatures, sleep was more disrupted, and heart rates increased [9]. In Detroit, Michigan, the thermal conditions of 30 different homes were monitored and analyzed, along with the housing characteristics (e.g., exterior wall materials). The findings showed that indoor exposure to heat in Detroit exceeded the comfort range among elderly occupants [12]. In the United States, there have been only a few studies on a larger scale of a single city (e.g., Detroit) [13], a single state (i.e., California) [14], or multi-county [15]. To the authors’ knowledge, there is no multistate study that has focused on the connection between heat-related illness and housing characteristics.

Indoor heat exposure potential is determined by the outdoor ambient temperature and housing characteristics (e.g., housing thermal inertia)..The majority of epidemiological studies of heat-related health effects use outdoor weather conditions as the primary indicator to estimate indoor heat exposure and/or heat stress [16,17,18]. Currently, most heat-health warning systems are also based on outdoor temperatures. This reliance on outdoor conditions can mislead the interpretation of health effects and associated solutions, since most people who stay indoors are assumed to be isolated from outdoor thermal conditions [19]. Currently, heat exposure in epidemiological studies is often estimated using an airport monitoring station and applied to residents of an entire community [12]. As for indoor heat exposure, a WHO working group on indoor environments found that “There is no demonstrable risk to human health of healthy sedentary people living in air temperature of between 18 and 24 °C” [20]. However, an adaptive thermal comfort model showed that thermal comfort also depends on other individual variables such as metabolism, level of activity, and clothing, among others. Therefore, a variety of indoor heat exposure ranges were found in previous literature. For example, 27 °C was used as the cut-off temperature in a survey of 57 elderly adults in the United States to study thermal conditions, reduced emotional distress, and increased hours of sleep [21]. Conversely, a study on 113 elderly people in the Netherlands used 20.8 to 29.3 °C as the temperature range, which led to similar conclusions that an increased temperature can raise the risk of sleep disturbance [22]. In the United States, there is no consensus on a cut-off maximum temperature for heat-related health risks; for instance, Boston uses 25 °C as an indoor maximum acceptable temperature, while New York City uses 27–28 °C [23]. Consequently, indoor heat exposure in this study should be understood as a range of higher temperatures over an extended period of two or more consecutive days.

Moreover, the Heat Vulnerability & Preparedness index provided by the US Centers for Disease Control and Prevention (CDC) considers 14 indicators including population demographic information and outdoor conditions, such as the percentage of forest canopy cover. However, there are no housing (building) indicators included. While outdoor weather conditions can be monitored or measured through multiple methods, such as ground monitoring, numerical models, and remote sensing data, indoor thermal conditions are not routinely monitored or reported due to privacy concerns and the time-consuming, labor-intensive traditional monitoring methods. Consequently, direct indoor heat exposure and heat stress are less studied than outdoor heat. While it is commonly perceived that buildings with little insulation, thermal mass, or shading are prone to overheating when air-conditioning is unavailable, supporting empirical studies are limited. Many practical models have been generated to predict indoor temperatures using outdoor temperatures, housing characteristics, and other variables. Some recent models, based on deep-learning computer algorithms, have reached a high accuracy of up to 98.4% [24]. However, there is a lack of direct methods for and evidence of connecting housing characteristics and indoor heat exposure with heat-related illness, which imposes difficulties in utilizing resilient building designs (e.g., passive design) to adapt to changing climate conditions [25]. To this extent, this study addresses these gaps by examining the association between housing characteristics and HRI.

The purpose of this study is to examine the correlation between housing characteristics and HRI at a national scale using data extracted from the American Housing Survey, the American Community Survey, the ResStock database, and the CDC’s National Environmental Public Health Tracking Network on a state level in the United States. More specifically, there are three questions addressed: (1) whether HRI can be predicted based on housing characteristic variables, (2) how influential these variables are, and (3) whether the variables influencing different HRI measure differently. Additionally, we hypothesized that states with higher housing thermal inertia quality have a better mitigation effect on HRI.

## 2. Influential Factors and Measured Outcomes

### 2.1. Housing Characteristics

A large body of epidemiological literature links general housing and living conditions to various health conditions, such as asthma and other respiratory diseases. While these are not direct HRI, prolonged heat exposure is linked to increased hospital admission for cardiovascular, kidney, and respiratory diseases [6].Therefore, this study includes general housing conditions, which are measured by two indicators: *housing age* and *housing size*. These two variables were used as a proxy measure of housing physical conditions.

Living conditions are included as a separate category. The *housing crowding ratio* and the *percentage of low-income housing* were used together as a proxy measure of residents’ living conditions. Overcrowding in housing and a lack of ventilation can promote a moist environment that leads to respiratory problems [26]. This threat is particularly high during the summer, in areas that are hot and humid, and when the air humidity is high [27,28]. In addition, living in crowded conditions can give rise to psychological distress that has a compounding effect on vulnerable populations during heat waves [29]. Crowding measures how many households have more occupants than rooms. According to the US Census Bureau, homes with more than 1.5 persons per room were counted as severely crowded, and homes with 1.01–1.5 persons were considered moderately crowded [30]. In this study, we aggregate severely crowded and moderately crowded homes.

From the limited epidemiological literature focusing on indoor heat exposure, we found that the following building characteristics are linked to heat-related morbidity and mortality: building age, prevalence of air-conditioning, and the thermal property of the exterior walls and roof [31,32,33]. In addition, according to the World Health Organization, cooling systems, building materials, and ventilation and shading devices are crucial factors that can mitigate indoor heat exposure through reducing indoor temperatures [23]. Conversely, in the building science and architectural design field, there is a large body of research on the effectiveness of reducing indoor temperatures through roof and exterior wall materials with a high thermal mass [34,35]. Therefore, in this study, housing thermal inertia is included as a separate category and has three indicators: *roof condition*, *exterior wall condition,* and *housing energy efficiency*. Thermal inertia is the measure of how well building materials and components can absorb solar heat without increasing the temperature [34], and it is largely influenced by the types of materials and insulations used in exterior walls and roofs. Roof thermal inertia and exterior wall thermal inertia are common variables that define thermal properties of buildings [36]. Residential buildings and small-scale commercial buildings are skin-load dominated buildings, where heat transfer is primarily determined by the influence of the exterior climate on a building’s envelope, or “skin.” The external walls and roof are important components of the building envelope. They allow passive control of indoor thermal conditions through the management of external heat transfer [37]. There is no available data on the thermal inertia value of housing on a state level; therefore, in this study, the roof and exterior wall conditions were used as proxy indicators for thermal inertia. The assumption is that roofs in poor condition (e.g., leaks, sagging, or holes) indicate a lower thermal inertia, with the same logic applied to the exterior walls. Housing energy efficiency is based on the relation between housing thermal property and energy consumption [38]. The United States did not employ a national model energy code for buildings until 1994 [39]. The model code specifies the thermal property of roofs and walls in different climate zones. Houses built before 1994 have potentially lower thermal properties, and most houses built before the 1970s did not contain any insulation [40]. During the summer, houses with a lower thermal inertia consume more energy (electricity) to cool the building. Consequently, housing energy efficiency can also be used to indicate the thermal inertia of houses.

In summary, built upon the results from the literature review of works from other publications in both built environment research and epidemiology fields, influencing housing characteristics can be grouped into three categories: general housing conditions, living conditions, and housing thermal inertia. Table 1 lists the factors and data sources of each category. Eight variables were used to index housing characteristics: (1) *housing age* (HA), (2) *housing size* (HS), measured in gross square footage; (3) *prevalence of air-conditioning (AC),* measured by the percentage of housing with air-conditioning units (including central and non-central systems)*;* (4) *housing crowding ratio* (HCR), the most common measure of overcrowding is persons per room in a dwelling unit; this study uses the U.S. Census Bureau’s definition (>1.5 persons per room as severely crowded, >1 persons per room as moderately crowded) [30]; (5) *percentage of low-income housing* (PH); (6) *roof condition* (RC), measured by the percentage of housing with roof problems, including a sagging roof, missing roofing material and a hole in the roof; (7) *exterior wall condition* (EWC), measured by the percentage of housing with exterior wall problems, including missing bricks, siding, or other outside materials, and sloping outside walls; and (8) *housing energy efficiency* (HEE), measured by site energy use intensity (kBtu/ft^2^). The percentage of the population that was 65 or older (A65) was used as a control variable. 

### 2.2. Measurement of Heat-Related Illness (HRI) 

Four measures were used to index HRI. Heat-related emergency department visits (EDV) is an age-adjusted rate of emergency department visits for heat stress per 100,000 population. It includes all cases where heat stress is listed as the primary diagnosis or one of the diagnoses. [27] The data were provided by state and/or local public health departments to CDC’s Environmental Public Health Tracking Program. These data represent the number of emergency department visits rather than the number of individuals. For example, a person visiting the emergency department twice in one year would count as two visits. Heat-related mortality (MORD) is the number of summertime (May–September) heat-related deaths over a five-year period (2015–2019). Based on data from death certificates, this indicator evaluates deaths that identified heat as an underlying or contributing cause. The data were suppressed if the number of deaths was less than 10. Heat-related hospitalizations (HOSP) is an age-adjusted rate of hospitalizations for heat stress per 100,000 population. Data were provided by state and/or local public health departments, and hospital admission records were selected using primary and other diagnosis codes. The heat-related mortality rate (MOR) is the ratio of MORD to the state population.

## 3. Method and Materials

**Figure 1:**
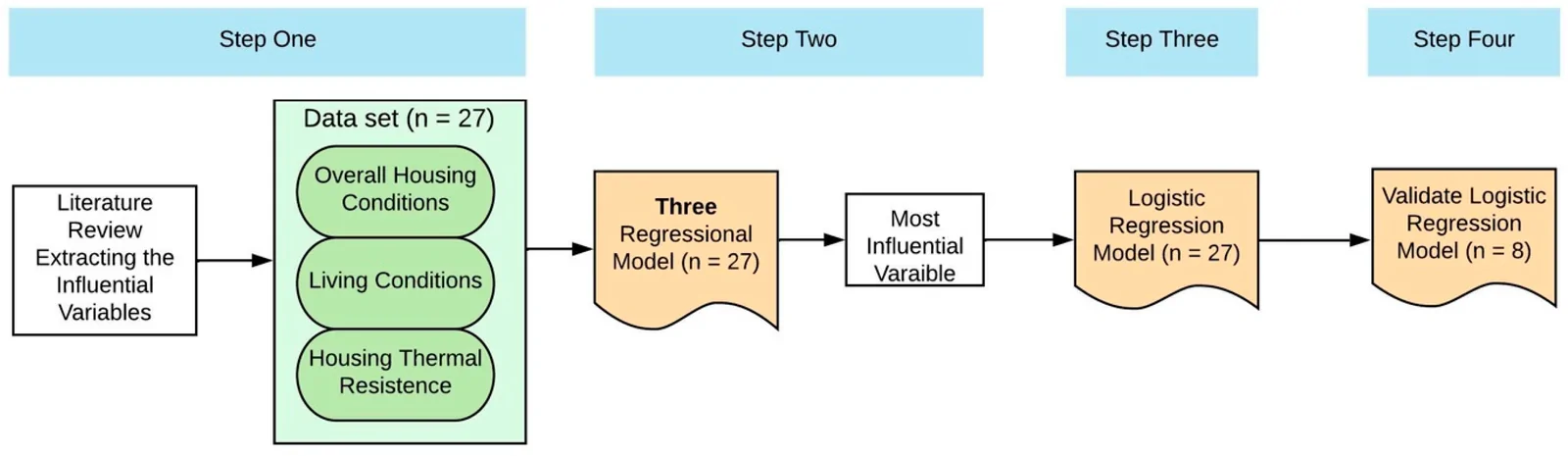
Research flow.

As illustrated in Figure 1, the research methodology of this study was composed of four steps. First, three categories and eight variables influencing the HRI index were identified from the literature review, and a data set containing data from 27 states was created. Second, three multivariable regression models of the individual HRI indexes were developed to determine the influential variables of each HRI index. Third, using the most influential variables identified in step two, a binary logistic regression model was generated to assess the risk of the HRI indexes on a state level. Fourth, the proposed logistic regression model was verified and validated using a data set with an additional eight states. 

### 3.1. Data Collection

Housing characteristic data were downloaded from three sources as listed in Table 1: the 2019 American Housing Survey (AHS), the ResStock database, and the American Community Survey. AHS is the most comprehensive national housing survey in the United States, which includes 3,494 variables related to housing characteristics. It has data on general housing conditions (e.g., size and age); rooms and amenities; heating, air-conditioning, and appliances; and housing qualities (e.g., roof condition). In addition, household demographics and low-income rental property data can be extracted from AHS [41]. Besides national data, AHS contained breakdown data of 11 states, the top 15 metropolitan areas (e.g., Boston-Cambridge-Newtown), and the next 20 metropolitan areas (e.g., Kansas City) [42]. Mobile houses were excluded in this study. Data on the *AC, PH, EWC,* and *RC* were extracted from AHS. The ResStock database is a housing stock characteristic database created and managed by the National Renewable Energy Lab based on the Residential Energy Consumption Survey [43]. It contains more granular information than the AHS; for example, it has breakdown information for housing types on state and county levels and detailed information on *HEE* per state and housing type. Data on *HA*, *HS*, and *HEE* (measured in kWh/m^2^/year) were extracted from ResStock. Data on *HCR* were extracted from America’s Health Rankings analysis based on the U.S. Census Bureau’s American Community Survey [29].

Data on the three HRI indexes—MORD, EDV, and HOSP—were extracted from CDC’s National Environmental Public Health Tracking Network on a state level. MOR was then calculated from MORD as described in Section 2.2. However, there were missing data on HRI measures. For example, CDC has MORD data for 36 states, EDV data for 27 states, and HOSP data for 31 states.

In our study, since complete and matching data on the HRI indexes and housing characteristic variables were needed, the 27 states’ data sets with the most complete information and data were used for regression model analysis and logistic model analysis. Additional missing HRI data on eight states were obtained from the Healthcare Cost and Utilization Project (HCUP). State-level data on EDV with a diagnosis directly indicating heat exposure were derived from the HCUP 2016–2020 State Emergency Department Databases (SEDD) and State Inpatient Databases (SID). The eight data sets were then used to test the proposed logistic model.

### 3.2. Statistical Analysis

First, a Pearson correlation matrix was created to understand the correlation coefficients between variables (refer to Table 2). The statistical significance of the correlation was determined to be *p* < 0.05; it appears as an asterisk (*) next to the correlation value. For example, the correlation between MORD and HA is 0.479; an asterisk means there is a statistically significant positive correlation between MORD and HA, and their correlation is moderate (between 0.3 and 0.69). A coefficient higher than 0.69 indicates a strong correlation, and HA and HEE have a correlation coefficient of 0.72. The variables with a statistically significant correlation were then used in the next step to create a regression model. As illustrated in Table 2, HA, HEE, and HCR are statistically correlated with MORD; HA and HEE are statistically correlated with MOR; and HA, HEE, RC, and AC are statistically correlated with EDV. Although EDV and MOR are correlated with HOSP, since this study focuses on the housing characteristics’ influence on HRI, this correlation among HRI measures was not further investigated in the regression model. The same logic was applied to the correlations among different housing characteristics. The further investigated variables are highlighted in Table 2. There were no housing characteristics statistically correlated with HOSP; therefore, HOSP was excluded in the second step for regression model analysis.

In step two, multivariable regression analysis was used to determine which variables in a model had a significant impact on the HRI index. After determining the influential predictors (significant variables), a multicollinearity test was used to determine the dependence of those variables. Variables that were highly dependent on other variables (VIF > 10 was used as a cut score) were ruled out. The multivariable regression analysis answers the following questions: (1) whether the HRI index is correlated with housing characteristic variables, (2) which housing characteristic variables are correlated with which HRI index, and (3) the relative influence of each variable on the variance in the HRI index. Three separate regression models were created and adjusted for A65 (refer to Equations (1)–(3)). MORD, EDV, and MOR were the dependent health outcomes.

For MORD,

(1) $$Y_{i}=\beta_{0}+\beta_{1}\left(HA\right)+\beta_{2}\left(HEE\right)+\beta_{3}\left(A65\right)+\mu_{i}$$

For EDV,

(2) $$Y_{i}=\beta_{0}+\beta_{1}\left(HA\right)+\beta_{2}\left(HEE\right)+\beta_{3}\left(RC\right)+\beta_{4}\left(AC\right)+\beta_{4}\left(A65\right)+\mu_{i}$$

For MOR,

(3) $$Y_{i}=\beta_{0}+\beta_{1}\left(HA\right)+\beta_{2}\left(HEE\right)+\beta_{3}\left(A65\right)+\mu_{i}$$

where $Y_{i}$ is the HRI index per state, $\beta_{1}$ to $\beta_{x}$ are the coefficients of variables, and $\mu_{i}$ is the random effect of intercept for the state.

The most influential variables identified from the regression model were then used to create the logistic model in step three. The difference between a logistic regression model and linear regression model is the dependent variable [44]. In the former, the dependent variable is binary or dichotomous. The logistic model created is illustrated in Equation (4). The goal of using a logistic model was to verify whether the HRI index could be predicted based on the identified housing variables (from the regression model).

(4) $$E\;\left(1/0\right)=\beta_{0}+\beta_{1}X_{1}+\beta_{2}X_{2}+\mu_{i}$$

where *E* denotes the possibility of a high risk in the HRI index (MORD, EDV, MOR): = 1 for a high risk and 0 = for a low risk. The threshold for determining the high and low risks is explained in Section 4.6 $\beta_{o}$ is the coefficient of the constant term, $\beta_{i}$ denotes a model parameter (the most influential variable), *X* is a value of the independent variable, and $\mu_{i}$ as is the error term. For testing the logistic model, we used the eight remaining states.

## 4. Results and Findings

The findings obtained by analyzing the influential housing variables, in relation to the HRI indexes in 27 states, are summarized in this section. First, a descriptive analysis of housing characteristics in those states is presented. Then, variables contributing to the three HRI indexes and their influence are discussed based on regression model results. Lastly, the validity and uncertainty of the housing characteristic variables in predicting the HRI index are discussed based on logistic model results.

### 4.1. Descriptive Statistics of Housing Characteristics

To better understand the housing characteristic variations among the states, the general housing conditions, living conditions, and housing thermal inertia are presented in Table 3. The housing types included in this study are single-family detached, single-family attached, multifamily 2–4 units, and multifamily ≥5 units. The definitions and typology of the housing types are used in the Residential Energy Consumption Survey and adopted by AHS. HA is measured by subtracting the average built year from 2021. For example, using the ResStock database, homes in Arizona built before 1940 account for 2% of the total state housing, while those built between 1940 and 1979 account for 32% and those built after 1979 account for 66%. Aggregating the average HA of each age bucket results in an average HA of 48 in Arizona. HS is the aggregated average HS in the state, and the HCR is defined as the percentage of occupied housing units with more than one person per room. RC is measured by the percentage of housing that has physical problems with the roof (e.g., sagging roof, missing roofing material, hole in the roof), and EWC is measured by the percentage of housing that has physical problems with an exterior wall (e.g., missing bricks, sidings, water leakage).

From Table 3, several observations can be made. Washington state has the largest average HS (63,461 ft^2^), one of the oldest housing stocks (63), and the least problems with roofs and walls, while Colorado has the smallest HS (13,636 ft^2^) and the third youngest housing stock (53). California has the highest HCR (8.2%), followed by New York (4.9%) and Arizona (4.3%). Pennsylvania has the highest problematic EWC (8%), followed by Kentucky (7%), Maine (7%), New Hampshire (7%), and Tennessee (7%). New Hampshire has the highest problematic RC (7%), followed by Iowa (6%), South Carolina (6%), and Tennessee (6%). Poor RC and EWC are an indication of housing with a low thermal inertia. HEE does not always follow the trend as housing ages, indicating that older housing does not necessarily equal poor thermal inertia. A higher HEE value denotes housing with less energy efficiency, while higher energy use during the summer signifies housing with a lower thermal inertia. For example, Minnesota has the lowest HEE (68.44 kBtu/ft^2^), which is two times lower than that of California (28.88 kBtu/ft^2^), while their respective housing ages are 57 and 56. Lastly, AC varies from 99% to 44%. Overall, there is no general pattern or trend that can be observed directly from the collected housing characteristic data.

### 4.2. Heat-Related Illness (HRI) Index

Table 4 lists the three HRI indexes on a state level. Two observations can be made. First, the death count does not directly relate to EDV; for example, Louisiana has the highest EDV (57.39) but a relatively low MORD (58), while Arizona has a median EDV (31.45) but the highest MORD (890). Second, despite both measuring heat-related deaths, MORD and MOR do not follow the same pattern. Except for Arizona ranking first for both MORD and MOR, the other states have different rankings in the two indexes. For instance, California has the second highest MORD but has a MOR in the lower quartile.

### 4.3. Regression Analysis: Emergency Department Visits (EDV)

General housing conditions and housing thermal inertia were predictors for state-level EDV (Prob > F < 0.05). As listed in Table 5, the value of Prob(F) (column 4) is the same as the p-value, and a value <0.05 shows the regression model has statistical significance. The correlation coefficient (column 7) indicates the strength of a relationship between two variables, where the higher the value, the stronger the relationship. The regression results show that 43.78% of EDV variability was explained by four combined variables: HA, HEE, RC, and AC. Among the four variables, only RC was statistically significant (*p* < 0.05), and all variables were independent of each other (VIF<10). The coefficients of the variables show that RC has a positive correlation with EDV, which indicates that worsening roof conditions lead to lower thermal inertia of housing and, consequently, more frequent EDV. HA has a negative correlation with EDV, which implies that aging buildings are not prone to heat-related stress. The negative correlation between HEE and EDV shows that energy efficiency is an indication of updated mechanical (including air-conditioning) systems, where the better functioning an air-conditioning system, the higher the mitigation function it plays in HRI. The overall interpretation of the regression model is that worsening roof conditions lead to a higher EDV. According to regression analysis results, RC was brought into the logistic model to predict EDV.

### 4.4. Regression Analysis: Heat-Related Mortality (MORD)

General housing conditions and living conditions were predictors for state-level MORD (Prob > F < 0.05). As listed in Table 6, the regression results show that the combined variables—HA, HEE, and HCR—produced a 48.33% MORD variance. Both HEE and HCR have a positive correlation with MORD, while HA has a negative correlation with MORD. Among the three variables, HA and HCR have a statistical significance (*p* < 0.05). The interpretation is that the younger the housing age, the more crowded living condition, and the higher MORD. For the correlation coefficient, HCR has a much higher influence compared to HA. According to the regression analysis results, HA and HCR were brought into the logistic model to predict MORD.

### 4.5. Regression Analysis: Heat-Related Mortality Rate (MOR)

General housing conditions and housing thermal inertia were not predictors for state-level MOR (Prob >F >0.05). As listed in Table 7, HEE and HA do not have statistical significance (*p* > 0.05). In addition, the R-squared value and coefficient level is low. These observations indicate that the combined influence of HA and HEE are not significant enough to predict MOR. Accordingly, no logistic model was proposed for MOR. 

### 4.6. Statistic Results: Logistic Model

The logistic regression model was used to verify the influence of the housing characteristic variables on the likelihood of a high risk of EDV and MORD. Based on the results of the multivariable regression analysis, the RC was identified and used to create the logistic regression model to predict the risk of EDV. HA and HCR were identified and used to predict the risk of MORD. In this step, we recoded the EDV, assuming EDV < 22 indicates a low-risk state, while EDV > 22 signifies a high-risk state. A threshold of 22 was used because it was the median EDV value of the 36 states with EDV data. MORD was also recoded using a cut-off value of 40, which is the median MORD value of the 36 states with MORD data. 

Table 8 demonstrates a statistical significance of the prediction of MORD using HA and HCR; the corresponding Prob > χ^2^ values are less than 0.05. A significance level of 0.05 indicates there is a 5% risk of falsely concluding that an association exists; therefore, we concluded that the logistic model for MORD had a statistical significance. A high risk of mortality was positively associated with HCR (OR = 60.70, 95% CI:1.23) and negatively related to HA (OR = 0.895, 95% CI:0.612), thus, HCR is more influential than HA. Table 8 also shows there is no statistical significance for the prediction of EDV using RC, since the corresponding Prob > χ^2^ values are more than 0.05. 

In this proposed logistic model, two predictors (HA and HCR) have a statistically significant association with the binary result of MORD. Therefore, after determining the significance, we then examined the classification table which is shown in Table 9. Of the 27 states, 13 were high-risk states and 14 were low-risk states. The logistical model accurately predicted 9 out of 13 high-risk states, and 11 out of 14 low-risk states. The aggregated accuracy of predication was 74.07%. These results indicate that the proposed logistic model with two predictors has a high success rate in predicting the risk of MORD on a state level. The interpretation is that the younger the housing, the more crowded the living conditions, and the higher the risk of MORD. Moreover, the accuracy of prediction for high-risk states (75%) was slightly higher than that of low-risk states (73.33%).

**Figure 2:**
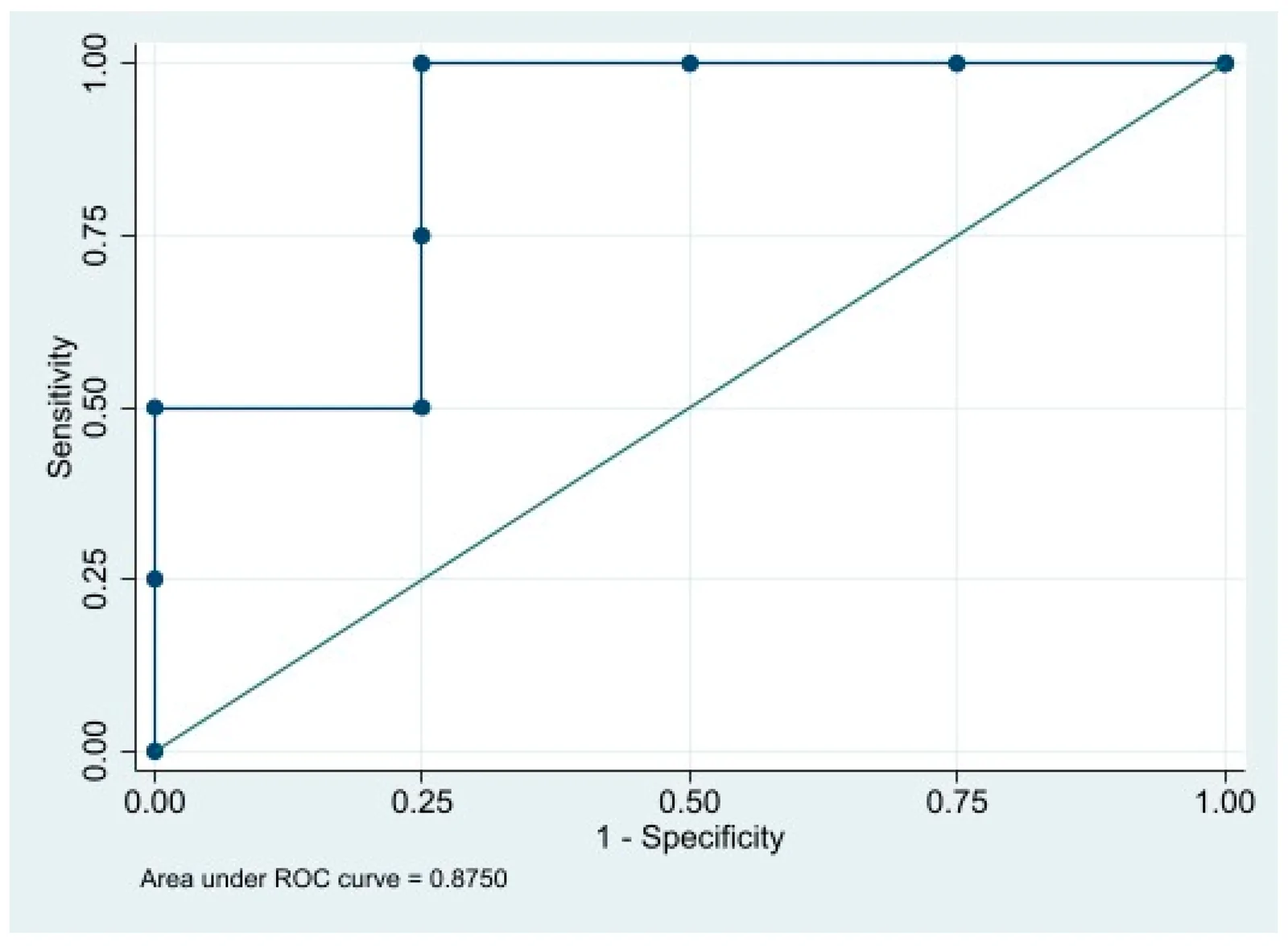
Logistic model for predicting the risk of heat-related mortality.

Lastly, we used the eight remaining states to test the logistic model. The model correctly predicted 100% of the high-risk states, but only correctly predicted 25% of the low-risk states. Figure 2 illustrates the test logistic model results; the ROC curve can reasonably predict the likelihood of a high-risk state for MORD, as the higher the ROC curve, the better the model fits the data. The area under the curve (AUC) is 0.875, larger than 0.5, which indicates that the model is much better than randomly estimating the outcome. A margin impact analysis on the logistic model was calculated to describe the average effect of changes in variables (HA and HRC) on changes in the probability of outcome (MORD), providing a direct and easily interpreted answer to the reliability of the logistic regression model [45]. For 10% of changes in HA and HRC, the probability of a high risk of MORD increased by 3.6%. The results showed that the effect of changes on these two housing characteristic variables on the high MORD is significant. Based on the limited testing, we can hypothesize that a combination of HA and HCR could be used to study the likelihood of high-risk states for MORD. Additional testing for a larger data set of states would be beneficial to refine and validate the model.

## 5. Conclusions

### 5.1. Study Contribution

Using a data set of 27 states, this study identified the correlation of housing characteristic variables in three categories, including general housing conditions, living conditions, and housing thermal inertia, with heat-related mortality (MORD), heat-related mortality rate (MOR), and heat-related emergency department visits (EDV). Out of the five identified influential variables (housing age (HA), housing energy efficiency (HEE), housing crowding ratio (HCR), roof condition (RC), and prevalence of air-conditioning (AC), RC has a statistical significance correlated with EDV; HA and HCR have a statistically significant correlation with MORD. The findings are discussed in three housing characteristic categories in the following sections.

The first two categories are closely related, with findings of the correlation between HA and HCR in line with previous studies. Moreover, the combination of these two variables can be used as predictors for the risk of MORD; these findings can help state agencies identify vulnerable communities and populations affected by extreme heat events. The regression model results of this study indicate that HA is negatively related to a higher risk of MORD. This differs from previous research and the common perception that older housing is linked to less thermal comfort. Findings from this study indicate that younger and newer housing may have less thermal inertia than older housing. This novel finding can be validated with additional data.

Although the individual correlations of HEE and AC were not found to be statistically significant to HRI, when combined with HA and HCR they were influential to MORD and EDV. This empirical evidence further supports the concept of the built environment determined health and climate change-exacerbated health outcomes.

The third category, housing thermal inertia (including RC), was not closely examined in previous research. This study contributes new findings on the role of housing thermal inertia in mitigating heat-related illness. The strong correlation between RC and EDV (from the regression model analysis) shows promise in mitigating heat stress by making roofs more thermally resistant. Relatively low-cost and low-tech solutions include adding additional insulation in the attic space or underside of the roof ceiling, or painting flat roofs in light colors and high reflective coating materials, which are readily available for most communities.

Other findings that do not align with previous research include the lack of correlation between the percentage of low-income housing (PH) and heat-related illness. This finding could help to dispel the perception that housing quality and thermal inertia equate to expensive construction. In addition, unlike RC, the exterior wall condition (EWC) did not correlate with any of the HRI indexes. There are two hypotheses: first, using an exterior condition as a proxy for thermal inertia is not reliable, and second, more indoor heat exposure is mitigated through the roof rather than the walls. Further data collection and analyses are needed to validate these hypotheses.

Overall, the results from this study provide useful insight, helping building owners and policy makers to make decisions or develop state-wide policies to support building upgrades or retrofits that adapt to extreme heat conditions. To the authors’ knowledge, this study is the first multistate study focusing on the connection between heat-related illness and housing characteristics. As climate change related, extreme heat events are projected to worsen for at least the next three decades [46], the findings from this study provide useful information to help health systems become more heat- resilient by integrating housing physical conditions as a mitigator. The results also reinforce the benefits of using data analytics to understand the correlation between housing characteristics and HRI. Findings of some less impactful variables are unexpected, but they are useful for providing direction for future studies.

### 5.2. Study Limitations

This study had four main limitations. First, our analysis data were limited to the state level. Data sets of 27 states were used for multivariable regression analysis and for building a logistic regression model. The limited data and samples may create selection bias and consequently affect the reliability of the analysis results. Therefore, the next step should be to expand the data set to include more states. Second, the selection of housing characteristic variables in this study may have influenced the findings. There are other housing variables besides the three categories in this study that impact HRI. To rule out other influencers, additional housing variables should be examined, especially variables contributing to the thermal inertia of housing, such as window (glass) areas that have direct exposure to sun and heat. A more in-depth literature review would help to extract additional variables to be included in this study. Third, data availability largely constrained the robustness of the analysis results. Since the data were collected and extracted from different sources, certain information did not match exactly. For example, EDV data from CDC was from 2015 to 2019, whereas EDV data extracted from HCUP was from 2016 to 2020. In addition, HRI data were not available for all states. It may be difficult to retrieve data for certain states with cold climates that have not tracked health data related to heat events, creating potential barriers for future research. Moreover, in the CDC database, there are no separate categories that differentiate outdoor heat-related data from indoor-heat-related data. The assumption used in this study is that during extreme heat conditions, most of the population is sheltered indoors. More granular and reliable data is needed for the specific purpose of studying indoor heat-related illness. To the authors’ knowledge, there is no such data set yet, which would be the next research step. Lastly, in this study, human factors were not included (e.g., activity level, underlying health conditions); in future studies, these variables can be used as control variables and be integrated into a regression model.

## 6. Conclusions

Heatwaves are the leading cause of death among weather events worldwide, in both developed and developing countries. The increase in extreme temperatures is expected to lead to a rise in heat related illness and deaths. Most research has focused on outdoor heat exposure and mitigation strategies, with studies on housing characteristics and their correlation with HRI being sparse. In this empirical study, three housing characteristic categories (general housing conditions, living conditions, and housing thermal inertia) and eight variables were analyzed using a multivariable regression model and a logistic model. Three variables (HA, HCR, and RC) were found to be correlated with a risk of HRI indexes. The logistic regression model was created using the three variables to predict the likelihood of the risk of EDV and MORD on a state level. The proposed model correctly predicted 100% of the high-risk states for MORD for the eight states tested. Overall, this analysis provides new evidence about the housing characteristic variables that influence HRI. The outcomes also reinforce the concept of built environment determined health, and demonstrate that the built environment, especially housing, should be considered as part of the techniques for mitigating climate change exacerbated health conditions. Those findings are useful for researchers from both the architectural engineering field and epidemiology field. 

## Figures and Tables

**Table 1:** Housing characteristic variables.

Factor	Abbreviation	Data Source
general housing conditions		
Housing age	HA	ResStock
Housing size	HS	ResStock
% Air-conditioning	AC	American Housing Survey
Living conditions		
% Low-income housing	PH	American Housing Survey
Housing crowd ratio	HCR	American Community Survey
housing thermal inertia		
Exterior wall condition	EWC	American Housing Survey
Roof condition	RC	American Housing Survey
Housing energy efficiency	HEE	ResStock
Control variables		
Age>65 ratio	A65	American Housing Survey

**Table 2:** Pearson correlation matrix.

	HRI Index				Housing Characteristics								
	MORD	HOSP	EDV	MOR	HA	HEE	HCR	(HS)	RC	(AC)	EWC	(PH)	A65
**MORD**	1												
**HOS**	0.349	1											
**EDV**	0.161	0.316	1										
**MOR**	0.924*	0.429*	0.261	1									
**HA**	−0.479 *	−0.228	−0.393 *	−0.45 *	1								
**HEE**	−0.457 *	−0.216	−0.431 *	0.35 *	0.72 *	1							
**HCR**	0.521 *	−0.089	−0.168	0.224	−0.04	−0.34	1						
**HS**	−0.143	−0.049	−0.107	−0.104	0.38 *	−0.07	0.01	1					
**RC**	0.526	0.015	0.497 *	0.086	−0.25	−0.16 *	−0.10	−0.05	1				
**AC**	0.138	0.026	0.392 *	0.140	−0.46 *	−0.12	−0.16	−0.49 *	0.29	1			
**EWC**	0.081	0.016	0.315	0.176	−0.22	0.07	−0.31 *	−0.20	0.72 *	0.43 *	1		
**PH**	−0.101	−0.100	−0.032	−0.009	0.47 *	0.43*	−0.11	0.08	−0.26	0.23	−0.10	1	
**A65**	−0.061	−0.061	−0.157	0.003	0.14	0.17	−0.23	−0.05	−0.11	0.15	−0.01	0.31	1

* significant values.

**Table 3:** Housing characteristic variables per state.

	General Housing Conditions			Living Conditions		Housing Thermal Inertia		
State	Housing Age (HA) Years	Housing Size (HS) ft^2^	Prevalence of Air-Conditioning (AC) %	Housing Crowding Ratio (HCR)	Percentage of Low-Income Housing (PH) *%*	Exterior Wall Condition (EWC) *%*	Roof Condition (RC) %	Housing Energy Efficiency (kBtu/ft^2^)
AZ	48	19,797	97%	2.73	30%	6%	4%	27.29
CA	56	17,823	76%	2.95	15%	4%	4%	28.88
**CO**	52	13,636	82%	2.65	10%	4%	2%	49.42
**CT**	60	28,280	63%	2.43	25%	4%	3%	54.50
**FL**	50	22,335	99%	2.48	14%	4%	4%	27.39
**IA**	63	22,448	98%	2.00	38%	6%	6%	59.92
**KY**	55	25,862	90%	2.61	18%	7%	5%	41.84
**LA**	54	20,955	99%	2.65	45%	4%	4%	28.66
**KS**	59	19,633	99%	2.46	37%	6%	4%	54.26
**ME**	59	19,340	94%	2.07	35%	7%	5%	67.85
**MD**	56	18,331	96%	2.59	41%	5%	4%	47.30
**MA**	63	26,025	87%	2.50	48%	3%	1%	60.06
**MI**	60	16,038	94%	2.34	46%	6%	4%	67.51
**MN**	57	21,483	97%	3.09	35%	6%	3%	68.44
**MO**	56	20,747	92%	2.39	17%	6%	5%	51.92
**NH**	57	32,970	87%	2.35	29%	7%	7%	55.60
**NJ**	60	21,624	52%	2.62	16%	4%	4%	55.63
**NY**	65	24,808	88%	2.54	52%	4%	3%	62.42
**NC**	51	17,869	99%	2.70	21%	4%	5%	31.91
**OR**	56	25,080	79%	2.71	29%	4%	2%	34.09
**PA**	56	25,080	92%	8.33	16%	8%	5%	34.09
**RI**	62	26,361	24%	2.40	25%	4%	3%	59.19
**SC**	51	17,599	93%	2.79	10%	6%	6%	29.07
**TN**	52	28,653	99%	2.65	31%	7%	6%	36.51
**VA**	53	21,072	98%	2.65	39%	4%	2%	41.08
**WA**	63	63,461	44%	2.79	30%	3%	3%	36.63
**WO**	60	25,041	94%	2.29	28%	4%	3%	64.18

**Table 4:** Heat-related illness (HRI) indexes.

	Heat-Related Illness Measures			
State	Heat-Related Mortality (MORD)	Heat-Related Mortality Rate (MOR)	Heat-Related Emergency Department Visits (EDV)	Heat-Related Hospitalizations (HOSP)
**AZ**	890	12.44	31.45	7.28
**CA**	369	0.93	13.50	1.76
**CO**	17	0.29	10.61	0.74
**CT**	45	1.25	13.01	12.70
**FL**	131	0.61	25.69	3.95
**IA**	23	0.72	33.56	1.46
**KY**	39	0.87	35.74	3.72
**LA**	58	1.25	57.39	3.64
**KS**	28	0.95	33.48	4.66
**ME**	9	0.66	17.50	0.87
**MD**	95	1.54	19.86	2.29
**MA**	9	0.13	10.80	1.27
**MI**	25	0.25	13.09	1.59
**MN**	28	0.32	14.69	1.27
**MO**	91	1.48	43.16	4.92
**NH**	9	0.65	14.46	0.87
**NJ**	26	0.28	12.30	1.48
**NY**	75	0.37	10.06	1.72
**NC**	66	0.63	24.96	1.40
**OR**	18	0.42	14.82	2.20
**PA**	85	0.65	15.00	0.84
**RI**	8	0.82	13.88	1.35
**SC**	67	1.31	35.40	3.90
**TN**	87	1.26	40.08	3.95
**VA**	58	0.67	13.73	3.00
**WA**	172	2.23	17.34	0.99
**WI**	134	2.27	11.69	1.18

**Table 5:** Regression analysis results: heat-related emergency department visits (EDV).

Regression Categories	Variables	R-Squared	Prob > F	t	*p*-Value	Coefficient
**General housing conditions**	HA	0.4378	0.0103 *	0.60	0.556	−0.442
	HEE			−1.86	0.079	−0.396
**Housing thermal inertia**	RC			2.24 *	0.036 *	328.30
	AC			1.01	0.134	19.90

* significant values.

**Table 6:** Regression analysis results: heat-related mortality (MORD).

Regression Categories	Variables	R-Squared	Prob > F	t	*p*-Value	Coefficient
**General housing conditions**	HA	0.4833	0.0014 *	−2.34 *	0.0928 *	−20.215
	HEE			−0.41	0.717	1.222
**Living conditions**	HCR			3.18 *	0.04 *	67.084

* significant values.

**Table 7:** Regression analysis results: heat-related mortality rate (MOR).

Regression Categories	Variables	R-Squared	Prob > F	t	*p*-Value	Coefficient
**General housing conditions**	HA	0.2024	0.0663	−1.61	0.122	−0.872
**Housing thermal inertia**	HEE			−0.16	0.874	−0.11

**Table 8:** Logistic regression model for heat-related mortality (MORD) and heat-related emergency department visits (EDV).

	Odds Ratio	*p*-Value	95% CI	LR χ^2^	Prob > χ^2^	Log Likelihood
***Heat-related mortality (MORD)***						
**HA**	0.895	0.04 *	0.612	6.28	0.0433 *	−2.153
**HCR**	60.698	0.006 *	1.23			
***Heat-related emergency department visits (EDV)***						
**RC**	767,000	0.022 *	790,820	7.73	0.0054	−14.832

* significant values.

**Table 9:** Classification table for logistic model results.

	Successful Predictions	Failed Predictions	Total # of States	Percentage Correct
**High Risk**	9	4	13	75%
**Low Risk**	3	11	14	73.33%
**Total**	12	15	27	74.07%

## Data Availability

The datasets generated during and/or analyzed during the current study are available from the corresponding author on reasonable request.
